# Efficacy and cardiovascular safety of the new estrogen-free contraceptive pill containing 4 mg drospirenone alone in a 24/4 regime

**DOI:** 10.1186/s12905-020-01080-9

**Published:** 2020-10-02

**Authors:** Santiago Palacios, Enrico Colli, Pedro-Antonio Regidor

**Affiliations:** 1 Instituto Palacios, Salud y Medicina de la Mujer, C/ Antonio Acuña 9, 28009 Madrid, Spain; 2 Exeltis HealthCare Madrid. C/ Manuel Pombo Angulo 28, 4thFloor, 28050 Madrid, Spain; 3 Exeltis Europe, Adalperostr, 84 85737 Ismaning, Germany

**Keywords:** Estrogen free contraception, Cardiovascular side effects, ECG, Progestogen, Spirolactone derivative, Drospirenone

## Abstract

**Background:**

A new estrogen-free contraceptive has been approved by both the FDA and more than 15 European authorities. It is composed of drospirenone (DRSP) at a dosage of 4 mg in a regimen 24/4. The molecule is known to have anti-gonadotropic, anti-mineralocorticoid, anti-estrogenic, and antiandrogenic properties. The purpose of these clinical trials with a new estrogen-free contraceptive was to introduce a contraceptive method with high efficacy and showing a profile with low cardiovascular risks.

**Methods:**

Three European and American multicenter clinical trials have been conducted in more than 2500 patients and more than 25,000 cycles, not only demonstrating an excellent efficacy (Pearl Index of 0.73) but also investigating possible cardiovascular risks. In the USA study, 422 participants (41.9%) had a risk factor for VTE, while in the European studies, 261 patients (16.6%) had at least one VTE risk factor. Amount of arterial and venous thromboembolic events, hemostasiological data, blood pressure development, and ECG data were evaluated.

**Results:**

No single case of VTE was documented, no changes in hemastosiological parameters were observed, a small decrease in RR in patients with pretreatment values between 130 and 140 and/or 85 to 90 mm HG and no influence on ECG parameters were observed.

**Conclusions:**

The introduction of a new estrogen-free contraceptive with 4 mg of non-micronized drospirenone in a 24/4-day regimen expands contraception options for women as not only a high efficacy could be demonstrated during clinical trials but also a very high cardiovascular safety profile was observed even in women with cardiovascular risk factors.

**Trial registration:**

EudraCT registration numbers: 2010–021787-15 & 2011–002396-42. Clincaltrials.gov: NCT02269241.

## Background

Combined hormonal contraceptives (CHC) are widely used, highly accepted, provide adequate protection, have a low side effect profile, and are associated with additional health benefits.

The main concerns regarding their use are cardiovascular risks – mainly the venous thromboembolism (VTE) risk – which on the one hand, make these preparations unsuitable for women with risk factors, but on the other hand, give rise to justified fears in healthy women [1].

In addition to this limitation, the increased risk of VTE events in healthy COC users is 6–12/10,000 women/year compared to a risk of 2/10,000 women/year in non-users. In the further subdivision, according to the latest dates of the EMA, the risks of the combined preparations with levonorgestrel or norethisterone are 5–7 cases/10,000 women/year, 8–11 cases in the ethinylestradiol-dienogest-containing and 9–12 cases in the combined formulations with the progestogenic desogestrel, gestodene and drospirenone [2].

This development was based on laboratory and epidemiological data suggesting that estrogen is the responsible component in the COC concerning an increased cardiovascular risk and that the progestogens per se (except for medroxyprogesterone acetate and norethisterone) do not increase this risk.

Laboratory data show that ethinylestradiol exerts a pro-coagulator effect due to its metabolism in the liver by increasing the factors responsible for clotting and reducing fibrinolytic factors. Estradiol or estradiol valerate is thought to have a lower influence in the liver than ethinylestradiol [3] due to shorter half-life and faster metabolization. Estrogens alter the dynamic balance of hemostasis by increasing coagulatory factors (e.g., factor VII) and anti-fibrinolytic factors (e.g., PAI-1). The number of D-dimers increases sequentially due to the higher content of fibrin and its degenerate products in the blood. This balance is also influenced by the amount of ethinylestradiol concentration that activates the coagulation and the sort and dose of the progestogen that enables anti-fibrinolytic factors such as PAI-1 [4].

As mentioned above, epidemiological studies have shown that COC increases the risk of VTE by two to four times, depending on the dose of estrogen and the type of progestogen [1, 2]. Progestogens per se do not increase the rate of thrombotic events, except for those with a partial glucocorticoid activity [4, 5].

The aim of the presented studies was to provide information regarding drospirenone (DRSP) 4mg (24/), and its cardiovascular safety, including women at high risk or with contraindications to the use of COC and to present data on efficacy and bleeding profile.

## Methods

Three multicentre phase III studies were performed to demonstrate the efficacy and safety of a DRSP only contraceptive pill in Europe and in the USA. These prospective studies were performed to obtain the market authorisation of the drospirenone only pill. The data of these three studies were used for the analysis of the primary endpoint; the overall PI. Also, the secondary endpoints were PI after correction for additional contraception for sexual activity status, method failure PI, and cumulative pregnancy rate.

In addition, a series of cardio-vascular risk factors like BMI, age, smoking status, etc. were documented and a series of clinical and laboratory examinations like RR measurement, ECG measurement and the obtention of hemostasiological parameters were evaluated.

In the European trial’s patients were enrolled in a total of 88 centres in Austria, Czech Republic, Germany, Hungary, Poland, Romania, Slovakia, and Spain. The studies were performed between July 11, 2011, and January 27, 2014. In the USA study, the patients were enrolled at 39 trial centres. The study was performed between October 09, 2014, and October 04, 2017.

### Ethics approval

For each of the investigational centres, ethical approval was obtained.

Each center’s Institutional Review Board approved the protocol. Women provided written informed consent prior to enrolment. Registration was at clincaltrials.gov: NCT02269241 for the USA trial and EudraCT registration numbers: 2010–021787-15 & 2011–002396-42 for the European trails.

### Study medication

In both European studies, the study medication was one tablet containing 4 mg non-micronized DRSP per day, via the oral route, with consecutive administration of 24 active pills and four placebo tablets, and no tablet-free interval between two successive cycles. The dosage was determined during phases I and II studies.

The patients used between 9 and 13 cycles the study medication. Urine-pregnancy tests were conducted at every study visit.

USA trial: Prospective, open-label, single-arm, multicentre trial. Participation included up to thirteen 28-day treatment cycles. Urine-pregnancy tests were conducted at every study visit. Participants also performed home urine-pregnancy tests at the start of each cycle.

During each cycle, participants swallowed one active tablet containing drospirenone 4 mg for 24 consecutive days, followed by four days placebo.

### Study populations

Inclusion criteria for the European studies were; women of child-bearing potential, at risk of pregnancy, agreeing to use only the study medication for contraception for the duration of the study treatment, aged 18 to 45, with systolic blood pressure (SBP) < 140 mmHg and diastolic blood pressure (DBP) < 90 mmHg.

#### USA trial

Sexually active, healthy, non-pregnant women aged ≥15 years seeking contraception were eligible for enrolment. Breastfeeding women at least six weeks postpartum could enrol for safety evaluations only. Non-menopausal participants had regular menstrual cycles during the previous six months when not using hormonal contraception or three complete cycles after birth if not breastfeeding. Participants could have prior experience using COCs.

### Statistical analyses

In each study, efficacy analyses were performed on the full analysis set (FAS), defined as all subjects who took at least one dose of the study medication and who were not pregnant at entry.

The primary and secondary efficacy endpoints were calculated separately for the European and the USA study and for both studies pooled, as well as their 95% Confidence Intervals (CI). Students t-test’s and Wilcoxon-rank-sum-tests were used for the recorded comparative cardiovascular risk factors.

## Results

Archer et al. [6], Palacios et al. [7] and Kimble et al. [8] showed in different clinical trials the contraceptive efficacy of drospirenone 4 mg. These trials were performed in the European Union (2 clinical phase III trials) and in the USA.

The primary endpoint of all trails was to obtain a satisfactory Pearl Index (PI). The pooled analysis of both European studies showed a total PI of 0.73 [95% CI: 0.3133; 1.4301] (14,329 cycles of drospirenone 4 mg) and an adjusted PI of 0.7898 [95% CI: 0.3410; 1.5562] [7].

In the USA clinical trial 17 pregnancies (Pearl index: 4.0 (95% confidence interval [CI], 2.3–6.4, *n* = 953), of which three were unconfirmed and two were from sites excluded from the main analysis for major breaches of FDA regulations were documented. These pregnancies were all detected in non-breastfeeding women aged ≤35 years. For confirmed pregnancies among 915 non-breastfeeding women aged ≤35 years from sites with no protocol violations the Pearl Index was 2.9 (95% CI: 1.5–5.1) [8].

### Bleeding profile

Findings in the European studies: A clear pattern to less bleeding during treatment was observed in both EU studies. In general, after 9–13 cycles of the use of DRSP approximately 40% of the users had an amenorrhoea. In one study a comparison between DRSP and desogestrel (DSG) was performed showing that in each cycle, up to cycle 7, the proportion of women with unscheduled bleeding including those which did not bleed was statistically significantly lower under the use of DRSP comparing it DSG (*p* = 0.0001, chi-square test). The mean [SD] number of unscheduled bleeding and spotting days during cycles 2–9 was 21.5 [22.86] days for DRSP vs. 34.7 [33.73] days for DSG, *p* = 0.0003, Wilcoxon-rank-sum-test. Unscheduled bleeding days between the cycle 2–6 were lower in the DRSP group compared to the DSG; excluding the amenorrhoeic women (p = 0.0001, chi-square test). The mean [SD] number of bleeding days was 8.6 [8.52] days vs. 12.9 [16.47] days, *p* = 0.0233 [9].

Findings in the USA study: Unscheduled bleeding was recorded by 187/523 women (45.5%) in the second cycle and this declined with continued use; approximately one-third (71/239) recorded unscheduled bleeding in Cycle 13. Over the time the mean duration of all bleeding and/or spotting episodes decreased accompanied with a trend towards fewer prolonged bleeding and/or spotting. Over time, a greater proportion of women reported amenorrhea [8]. About one-third of participants (169/523; 32.3%) reported scheduled withdrawal bleeding in the second cycle and the frequency declined with continued use.

### Cardiovascular safety

#### Hemostasiological parameters

Thirty-nine patients taking drospirenone 4 mg (24/4) and 29 patients taking desogestrel 0.075 mg daily as a comparative group, over a continuous period of 9 cycles, were evaluated. The measured hemostasiological parameters were APC resistance, antithrombin III (ATIII), D-dimer, C-reactive protein (CRP), and clotting factors VII and VIII [10].

The clotting factor VII (FVII) baseline value was lower in the drospirenone group (1.12, SD 0.2486) than in the desogestrel group (1.24, SD 0.2607). The FVII averages were comparable between the two groups, but the difference from the starting point- to the end value was with drospirenone 4 mg (difference statistically significant), more pronounced (*p* = 0.0088). The mean C-reactive protein (CRP) baseline value in the drospirenone group (1.14, SD 0.2052) was also lower than in the desogestrel group (1.29, SD 0.2447, *p* = 0.0069). The changes in the final values were identical (1.10, SD 0.1688) for drospirenone vs. desogestrel (1.13, SD 0.2230). The difference in the mean change from starting value to final value was 0.03 for drospirenone vs. 0.15 for desogestrel (*p* = 0.0249). A significant D-dimer reduction was observed in the drospirenone group. The baseline values were 264.9 ng/ml and fell to 215.0 ng/ml; while in the group of desogestrel, there was an increase from 201.4 ng/ml to 281.5 ng/ml (see Table 1). The observational period was 13 complete cycles and the laboratory examinations were performed just shortly before starting the medication and at day 29 + 2 of the last cycle. Exams were done once before and after the study.

**Table 1:** Baseline and endpoint values of hemastosiological parameters

	APC resistance	ATIII	FVII	FVIII	Protein C	D-dimer (ng/ml)
Baseline	2.711	0.946	1.123	0.939	1.14	264.9
Endpoint	2.998	0.99	1.066	1.012	1.108	215

#### Thromboembolic events

During the clinical development program of drospirenone 4 mg, there were no reports of venous thromboembolism (VTE). There were also no reports of arterial thromboembolism, myocardial infarcts, strokes, or pulmonary embolisms.

In phase III clinical trials 1, 2, and 3 (Europe and US studies), a significant number of participants with risk factors for VTE have been enrolled. In the US, 422 participants (41.9%) had a risk factor for VTE, while in studies 1 and 2, 116 patients (16.3%) had a risk factor and 145 (16.9%) respectively. See Tables 2 and 3.

**Table 2:** Patients characteristics of thromboembolic risk factors of the European studies

		Study 1	Study 2	
n (%)		Drospirenone	Drospirenone	Desogestrel
		(*N* = 713)	(*N* = 858)	(*N* = 332)
Age, mean (SD), yr		28.7 (7.1)	28.9 (7.1)	28.9 (7.1)
Age group	≤35 yr	569 (79.8)	682 (79.5)	259 (78.0)
	> 35 yr	144 (20.2)	176 (20.5)	73 (22.0)
BMI, mean (SD) (kg/m2)		23.0 (3.8)	23.0 (3.5)	22.8 (3.9)
BMI group	< 30	672 (94.2)	828 (96.5)	316 (95.2)
	≥30	41 (5.8)	30 (3.5)	16 (4.8)
BP group (mm Hg)	SBP < 130, DBP < 85	571 (80.1)	727 (84.7)	290 (87.3)
	SBP ≥130, DBP ≥85	142 (19.9)	131 (15.3)	42 (12.7)
Presence of ≥1 VTE risk factor		110 (15.4)	142 (16.5)	59 (17.8)
Current smoker		182 (25.5)	237 (27.6)	103 (31.0)
Regular menstrual bleeding during the last 6 cycles		680 (95,4)	786 (91.6)	305 (91.9)
Prior treatment with sex hormones and modulators of genital system		455 (63,8)	704 (82.1)	288 (86.7)
Starters		287 (40.3)	417 (48.6)

**Table 3:** Patients characteristics of thromboembolic risk factors of the USA study

Risk factors		*N* = 1006
Family history of thromboembolic illness, n (%)	Yes	12 (1.2%)
	No	993 (98.8%)
	Missing	1
Evidence of predisposing conditions for a vascular or metabolic disease, n (%)	Yes	5 (0.5%)
	No	1001 (99.5%)
Current smoker older than 35 years or non-smoker over 40 years old, n (%)	Yes	51 (5.1%)
	No	955 (94.9%)
BMI > 30 kg/m^2^,n (%)	Yes	353 (35.1%)
	No	653 (64.9%)
Number of VTE risk factors, n (%)	0	611 (60.8%)
	1	367 (36.5%)
	2	27 (2.7%)
	≥3	0
	Missing	1

The main risk factors were age > 35 years and BMI > 30. BMI values over 30 were present in 71 patients in Europe and 388 in the USA. There, 188 women had a BMI > 35, and 88 had a BMI > 40.

A further risk factor was smoking. In 26% of the cases, smokers were randomized in the EU and 18% % in the US study.

Afro-American and Hispanic women who per se have a higher thromboembolic represented 35.6 and 22.8% of the population in the USA trial.

These data are consistent with the hemostasiological parameters of drospirenone 4 mg [10]. The observational period to assess these events were between the first visit at study entry and at day 29 + 2 of the last cycle.

#### Effects on mild hypertension

The administration of drospirenone in combination with estrogens for six months is associated with a slight decrease in systolic (SBP) and diastolic (DBP) blood pressure, compared to levonorgestrel in combined formulations [11]. This small influence on blood pressure was also shown when 3 mg drospirenone was compared with 0,015 mg desogestrel [12]. These results are justified by the anti-mineralocorticoid effect of drospirenone.

In study 1 in Europe, an average decrease of 8 mmHg in systolic blood pressure (SBP) and 5 mmHg in diastolic blood pressure (DBP) was observed in participants who had baseline values > SBP 130 mmHg or > DBP 85 mmHg (*n* = 137). In the group with average SBP (< 130 mmHg) and/or DBP < 85 mmHg (*n* = 548), the absolute mean change was 0.00 mmHg for SBP and DBP (see Table 4).

**Table 4:** Blood pressure development of study 1 in Europe

		SBP < 130 and DBP < 85 (mmHg)	SBP ≥ 130 and DBP ≥ 85 (mmHg)
Changes from baseline		N = 548	N = 137
SBP (mmHg)	Mean (SD)	1.77 (10.08)	−7.59 (9.19)
	**Median**	**0.0**	**−8.0**
DBP (mmHg)	Mean (SD)	1.06 (8.20)	−4.85 (7.85)
	**Median**	**0.0**	**−5.0**

The data in study 2 showed that the women with a baseline value of SBP > 130 mmHg or DBP > 85 mmHg (*n* = 130) had an average decrease of 7.0 mmHg with drospirenone 4 mg for the systolic value and 5.5 mmHg for the diastolic value over time. For participants with average SDP (< 130 mmHg) and DBP < 85 mmHg, the absolute mean change was 0.00 mmHg for both parameters.

The observational period to assess these events were between the first visit at study entry and at day 29 + 2 of the last cycle. Exams were performed at each visit (cycle) during the study.

#### Electrocardiogram (ECG)

The following variables related to ECG were collected for 151 women using DRSP at the day starting with the study medication (visit 1) and after finishing the study (at day 29 + 2 of the last cycle) (visit 2) and 56 women using desogestrel as a control group. These parameters were evaluated: (mean) heart rate, RR, PR, and QRS duration, QT duration, QTcB – Bazett’s correction formula, and QTcF-Fridiricia’s correction formula. These ECGs were centrally evaluated.

At the starting visit summary, mean (SD) QRS duration was comparable between the treatment groups: 90.9 (8.1) ms in the drospirenone and 89.6 (8.3) ms in the desogestrel group. Statistically significant differences in mean (SD) QRS duration between the treatment groups were seen at visit 2: 92.0 (8.4) ms in the drospirenone vs. 88.4 (8.6) ms in the desogestrel group (LS mean difference of 3.58 ms, 90% CI: 1.40; 5.77) and with regard to the mean (SD) change from visit 1 to visit 2: 1.5 (5.4) ms in the drospirenone vs. -1.1 (5.0) ms in the desogestrel group (LS mean difference of 2.55 ms, 90% CI: 1.13; 3.97). Nevertheless, these differences were not significant.

In the drospirenone group, the mean (SD) QT interval changed from 383 (22.1) ms at visit 1 to 390.8 (23.0) ms at visit 2. In the desogestrel group, it changed from 385.3 (19.9) to 384.3 (27.1). No statistically significant differences at both visits were observed between the treatment groups. The mean (SD) QT duration changed by 8.0 (22.0) ms in the drospirenone group and by − 0.9 (20.5) ms in the desogestrel group. The LS mean difference of 8.90 ms between the groups was statistically significant (90% 2-sided CI: 3.13; 14.68).

The mean (SD) QTcB interval increased by 0.7 (15.8) ms in the drospirenone group and decreased by − 1.5 (20.7) ms in the desogestrel group, and the difference of 2.19 ms between the groups’ changes was not statistically significant (90% 2-sided CI: − 2.44; 6.82).

The mean QTcF interval increased by 3.2 (12.3) ms in the drospirenone group and decreased by − 1.3 (14.5) ms in the desogestrel group. The difference of 4.55 ms between the groups’ changes was statistically significant (90% 2-sided CI: 1.09; 8.02). However, such change (4.55 ms) is below the threshold of regulatory concern (around 5 ms) given that it is not associated with an increased risk of torsade de pointes according to the Note for Guidance on the Clinical Evaluation of QT/QTc Interval Prolongation and Proarrhythmic Potential for Non-Antiarrhythmic Drugs [13].

The analysis of categorical QTcF indices demonstrated that in the drospirenone group one woman at visit one and at visit 2 had QTcF > 450 ms and one woman had a QTcF increase of 30 ms–60 ms from baseline at visit 2, whereas none of the women met these criteria in the desogestrel group. There were no women in either treatment group with QTcF > 470 ms or an increase from baseline of the QTcF > 60 ms. None of the drospirenone group women at visit 2 had a heart rate of < 50 bpm while one woman met this criterion in the desogestrel group. Table 5 shows the data.

**Table 5:** ECG data of 151 women before and after 9 cycles of 4 mg drospirenone

Parameter	Visit		Drospirenone	Desogestrel	LS Mean Difference 90% CI
**Summary (mean)**	Visit 1b	n	151	56	
**Heart rate (bpm)**		Mean (SD)	72.9 (9.4)	72.6 (10.1)	0.31 [−2.17; 2.80]
		Median	73.0	71.5	
		Min/ Max	51/106	49/102	
	Visit 5/EDV	n	138	58	
		Mean (SD)	70.7 (10.7)	72.7 (12.3)	−1.97 [−4.86; 0.92]
		Median	71.0	70.5	
		Min/ Max	50/102	49/104	
	Change from V1b	n	132	54	
		Mean (SD)	−2.5 (10.3)	0.3 (11.7)	−2.81 [−5.67; 0.05]
		Median	−3.0	0.0	
		Min/ Max	−34/18	−28/32	
**Summary (mean)**	Visit 1b	n	151	56	
**RR Duration**		Mean (SD)	830.8 (109.1)	836.5 (117.2)	−5.66 [−34.43; 23.11]
**(ms)**		Median	821.0	832.5	
		Min/ Max	564/1162	586/1223	
	Visit 5/EDV	n	138	58	
		Mean (SD)	861.7 (133.4)	842.0 (139.3)	19.68 [−15.27; 54.64
		Median	835.0	843.0	
		Min/ Max	586/1200	572/1218	
	Change from V1b	n	132	54	
		Mean (SD)	35.5 (121.8)	2.3 (125.9)	33.19 [0.33; 66.04]
		Median	29.5	0.5	
		Min/ Max	−198/391	− 306/260	
**Summary (mean)**	Visit 1b	n	151	56	
**PR duration (ms)**		Mean (SD)	150.0 (26.0)	150.2 (18.7)	−0.22 [−6.50; 6.06]
		Median	147.0	150.0	
		Min/ Max	91/305	115/207	
	Visit 5/EDV	n	138	58	
		Mean (SD)	151.1 (21.4)	150.8 (17.8)	0.35 [−4.93; 5.63]
		Median	149.5	152.5	
		Min/ Max	105/242	111/198	
	Change from V1b	n	132	54	
		Mean (SD)	−0.6 (17.6)	− 0.2 (11.6)	
		Median	0.0	0.0	−0.41 [−4.72; 3.90]
		Min/ Max	−117/73	−41/35	
**Summary (mean)**	Visit 1b	n	151	56	
**QRS duration**		Mean (SD)	90.9 (8.1)	89.6 (8.3)	1.31 [−0.79; 3.42]
**(ms)**		Median	90.0	89.0	
		Min/ Max	74/118	70/107	
	Visit 5/EDV	n	138	58	
		Mean (SD)	92.0 (8.4)	88.4 (8.6)	3.58 [1.40; 5.77]
		Median	92.0	87.5	
		Min/ Max	71/115	68/106	
	Change from V1b	n	132	54	
		Mean (SD)	1.5 (5.4)	−1.1 (5.0)	2.55 [1.13; 3.97]
		Median	1.0	0.0	
		Min/ Max	−13/20	− 12/8	
**Summary (mean)**	Visit 1b	n	151	56	
**QT duration (ms)**		Mean (SD)	383.3 (22.1)	385.3 (19.9)	−1.96 [−7.52; 3.60]
		Median	382.0	384.5	
		Min/ Max	332/437	335/438	
	Visit 5/EDV	n	138	58	
		Mean (SD)	390.8 (23.0)	384.3 (27.1)	6.50 [0.22; 12.78]
		Median	389.0	387.0	
		Min/ Max	330/454	317/442	
	Change from V1b	n	132	54	
		Mean (SD)	8.0 (22.0)	−0.9 (20.5)	8.90 [3.13; 14.68]
		Median	6.0	3.5	
		Min/ Max	−43/70	−49/51	
**Summary (mean)**	Visit 1b	n	151	56	
**QTcB - Bazett’s**		Mean (SD)	421.5 (17.3)	422.8 (20.5)	−1.33 [−6.05; 3.38]
**Correction Formula**		Median	420.0	420.5	
**(ms)**		Min/ Max	377/473	382/466	
	Visit 5/EDV	n	138	58	
		Mean (SD)	422.7 (19.2)	420.6 (17.5)	2.14 [−2.70; 6.98]
		Median	423.0	418.5	
		Min/ Max	373/483	392/463	
	Change from V1b	n	132	54	
		Mean (SD)	0.7 (15.8)	−1.5 (20.7)	2.19 [−2.44; 6.82]
		Median	−1.0	−5.0	
		Min/ Max	−44/40	−41/61	
**Summary (mean)**	Visit 1b	n	151	56	
**QTcF - Fridericia’s**		Mean (SD)	408.0 (14.7)	409.5 (15.1)	−1.56 [−5.40; 2.27]
**Correction Formula**		Median	407.0	410.0	
**(ms)**		Min/ Max	374/461	370/436	
	Visit 5/EDV	n	138	58	
		Mean (SD)	411.4 (14.5)	407.7 (14.8)	3.69 [−0.09; 7.47]
		Median	410.5	407.0	
		Min/ Max	371/458	381/450	
	Change from V1b	n	132	54	
		Mean (SD)	3.2 (12.3)	−1.3 (14.5)	4.55 [1.09; 8.02]
		Median	4.0	−2.0	
		Min/ Max	−28/48	−30/43	

## Discussion

No single case of VTE was documented, no changes in hemastosiological parameters were observed, a small decrease in RR in patients with pretreatment values between 130 and 140 and/or 85 to 90 mmHg and no influence on ECG parameters were observed.

Previous authors have described that the use of combined contraceptives accelerate the cascaded of coagulation and fibrinolysis (Kuhl [14], Winkler [15], and Schindler [16]). They could describe an increasing of various markers of hemostasis and fibrin turnover as a result of the pro coagulatoric effects of oestrogens and especially ethinyl oestradiol. The action of ethinylestradiol on hepatic and vascular function is well documented by the rise of sex hormone-binding globule (SHBG) [16]. In the combined drugs (COC) progestogens with pronounced androgenic properties, e.g., levonorgestrel, may counteract estrogen-induced changes in the hepatic synthesis of hematological factors. Other progestogens with antiandrogenic properties or with neutral androgenetic properties may not [16].

DRSP exhibits a different pharmacokinetic profile when administered together with ethynyl estradiol. While the new formulation with 4 mg DRSP contains 33% more active ingredient than a reference combined oral contraceptive (3 mg DRSP + 0·02 mg ethinylestradiol), the extent of systemic exposure at steady-state is using dose corrected data about 33% less with the new formulation, without dose correction (AUC_0-24h_, ss GMR = 77·81, 90% CI = 74·64% - 81·12%) [17]. Combined with a reduced C_max_, this pharmacokinetic profile of the new formulation may be relevant for similar efficacy and enhanced safety, both characteristics explaining the high efficacy and safety profile found in these clinical trials.

The shown parameters of haemostasiology, RR value development and ECG show that the new estrogen free contraceptive is not only a valid alternative for healthy women but also for specials collectives like overweight or obese women. Women with obesity have physiological changes compared to normal weight individuals, such as modifications in the cardiac output or alterations of the liver enzymes functions. Some of these changes have the potential to affect the absorption, distribution, metabolism and elimination of drugs, which may affect its effectiveness [18]. This is not the case under the use of the estrogen free contraceptive DRSP.

The estrogen-free contraceptive containing 4 mg of drospirenone in a 24/4 regimen intake provides effective contraception with a good safety/tolerability profile in a broad group of women, including overweight or obese women and is an option for most women regardless of blood pressure, BMI or thromboembolic risks.

## Data Availability

The datasets used and/or analysed during the current study available from the corresponding author on reasonable request.

## Abbreviations

FDA: Food and Drug Administration
DRSP: Drospirenone
DSG: Desogestrel
PI: Pearl Index
VTE: Venous Thromboembolism
RR: Riva Rocci
mm HG: Millimeter Hydrargyrum
ECG: Electrocardiogram
SBP: Systolic Blood Pressure
DBP: Diastolic Blood Pressure
PR: Puls Rate
QRS: Series of deflections in an electrocardiogram that represent electrical activity generated by ventricular depolarization prior to contraction of the ventricles
QT: QT Interval
QTcF: QT Interval corrected by Frequency
QtcB: Qt interval corrected by Bazett’s
SD: Standard Deviation
CHC: Combined Hormonal Contraceptives
COC: Combined Oral Contraceptives
BMI: Body Mass Index
AUC: Area Under the Curve
GMR: Geometric Median Ratio
EDV: Early Discontinuation Visit

## References

[1] WHO Medical eligibility criteria for contraceptive use. 5^th^ edition 2015. 26447268

[2] PRAC referral assessment report. Procedure under Article 31 of Directive 2001/83/EC resulting from pharmacovigilance data. Combined hormonal contraceptives containing medicinal products INN: chlormadinone, desogestrel, dienogest, drospirenone, etonogestrel, gestodene, nomegestrol, norelgestromin or norgestimate. Procedure number: EMEA/H/A-31/1356. October 15 2013.

[3] Schindler AE, Campagnoli C, Druckmann R, Huber J, Pasqualini JR, Schweppe KW, Thijssen JH (2008). Classification and pharmacology of progestins. Maturitas..

[4] Schindler AE (2013). Non-contraceptive benefits of Oral hormonal contraceptives. Int J Endocrinol Metab.

[5] Regidor PA, Schindler AE (2017). Antiandrogenic and antimineralocorticoid health benefits of COC containing newer progestins: dienogest and drospirenone. Oncotarget.

[6] Archer DF, Ahrendt HJ, Drouin D (2015). Drospirenone-only oral contraceptive: results from a multicenter noncomparative trial of efficacy, safety and tolerability. Contraception..

[7] Palacios S, Colli E, Regidor PA. Multicenter, phase III trials on the contraceptive efficacy, tolerability and safety of a new drospirenone only pill. Acta Obstet Gynecol Scand. 20198. doi: 10.1111/aogs.13688. 10.1111/aogs.13688 PMC7186823 31321765

[8] Kimble T, Burke AE, Barnhart KT, Archer DF, Colli E, Westhoff CL. A 1-year prospective, open-label, single-arm, multicenter, phase 3 trial of the contraceptive efficacy and safety of the oral progestin-only pill drospirenone 4 mg using a 24/4-day regimen. Contracept X. 2020;2:100020. 10.1016/j.conx.2020.100020. 10.1016/j.conx.2020.100020 PMC7286157 32550535

[9] Palacios S, Colli E, Regidor PA (2020). Bleeding profile of women using a drospirenone only pill 4 mg over nine cycles in comparison with desogestrel 0.075 mg. PLoS ONE.

[10] Regidor PA, Colli E, Schindler AE (2016). Drospirenone as estrogen-free pill and hemostasis: coagulatory study results comparing a novel 4 mg formulation in a 24 + 4 cycle with desogestrel 75 μg per day. Gynecol Endocrinol.

[11] Oelkers W, Foidart JM, Dombrovicz N, Welter A, Heithecker R (1995). Effects of a new oral contraceptive containing an antimineralocorticoid progestin, drospirenone, on the renin-aldosterone system, body weight, blood pressure, glucose tolerance, and lipid metabolism. J Clin Endocrinol Metab.

[12] Gaspard U, Scheen A, Endrikat J, Buicu C, Lefebvre P, Gerlinger C, Heithecker R (2003). A randomized study over 13 cycles to assess the influence of oral contraceptives containing ethinylestradiol combined with drospirenone or desogestrel on carbohydrate metabolism. Contraception..

[13] ICH Topic E 14 the clinical evaluation of QT/QTc interval prolongation and Proarrhythmic potential for non-antiarrhythmic drugs. European Medicines Agency. 2005. CHMP/ICH/2/04. 16237860

[14] Kuhl H (1996). Effects of progestogens on haemostasis. Maturitas.

[15] Winkler UH (1996). Hormone replacement therapy and haemostasis: principles of a complex interaction. Maturitas.

[16] Schindler AE (2003). Differential effects on progestins on haemostasis. Maturitas.

[17] Richter WH, Koytchev R, Kirkov V, Merki G, Colli E, Regidor P-A (2020). Comparative pharmacokinetic estimates of Drospirenone alone and in combination with Ethinyl estradiol after single and repeated Oral Administration in Healthy Females. Contraception.

[18] Ng M, Fleming T, Robinson M, Thomson B, Graetz N, Margono C, Mullany EC, Biryukov S, Abbafati C, Abera SF, Abraham JP, Abu-Rmeileh NM, Achoki T, FS AB, Alemu ZA, Alfonso R, Ali MK, Ali R, Guzman NA, Ammar W, Anwari P, Banerjee A, Barquera S, Basu S, Bennett DA, Bhutta Z, Blore J, Cabral N, Nonato IC, Chang JC, Chowdhury R, Courville KJ, Criqui MH, Cundiff DK, Dabhadkar KC, Dandona L, Davis A, Dayama A, Dharmaratne SD, Ding EL, Durrani AM, Esteghamati A, Farzadfar F, Fay DF, Feigin VL, Flaxman A, Forouzanfar MH, Goto A, Green MA, Gupta R, Hafezi-Nejad N, Hankey GJ, Harewood HC, Havmoeller R, Hay S, Hernandez L, Husseini A, Idrisov BT, Ikeda N, Islami F, Jahangir E, Jassal SK, Jee SH, Jeffreys M, Jonas JB, Kabagambe EK, Khalifa SE, Kengne AP, Khader YS, Khang YH, Kim D, Kimokoti RW, Kinge JM, Kokubo Y, Kosen S, Kwan G, Lai T, Leinsalu M, Li Y, Liang X, Liu S, Logroscino G, Lotufo PA, Lu Y, Ma J, Mainoo NK, Mensah GA, Merriman TR, Mokdad AH, Moschandreas J, Naghavi M, Naheed A, Nand D, Narayan KM, Nelson EL, Neuhouser ML, Nisar MI, Ohkubo T, Oti SO, Pedroza A, Prabhakaran D, Roy N, Sampson U, Seo H, Sepanlou SG, Shibuya K, Shiri R, Shiue I, Singh GM, Singh JA, Skirbekk V, Stapelberg NJ, Sturua L, Sykes BL, Tobias M, Tran BX, Trasande L, Toyoshima H, van de Vijver S, Vasankari TJ, Veerman JL, Velasquez-Melendez G, Vlassov VV, Vollset SE, Vos T, Wang C, Wang X, Weiderpass E, Werdecker A, Wright JL, Yang YC, Yatsuya H, Yoon J, Yoon SJ, Zhao Y, Zhou M, Zhu S, Lopez AD, Murray CJ, Gakidou E (2014). Global, regional, and national prevalence of overweight and obesity in children and adults during 1980-2013: a systematic analysis for the Global Burden of Disease Study 2013. Lancet.

